# Efficacy of a vaginal tablet as a Persian medicine product on vulvovaginal candidiasis: a double-blind, randomised, placebo-controlled trial

**DOI:** 10.1080/13880209.2020.1784236

**Published:** 2020-07-02

**Authors:** Somayyeh Khalilzadeh, Tahereh Eftkhar, Laila Shirbeigi, Malihe Tabarrai, Tayebeh Toliyat, Shamim Fayazmanesh, Zeinab Ghasemi, Safar Shamohammadi

**Affiliations:** a Department of Persian Medicine, School of Persian Medicine, Tehran University of Medical Sciences, Tehran, Iran; b Department of Obstetrics and Gynecology, Tehran University of Medical Sciences, Tehran, Iran; c Department of Pharmaceutics, Faculty of Pharmacy, Tehran University of Medical Sciences, Tehran, Iran; d Department of Pharmacognosy, Faculty of Pharmacy and Persian Medicine and Pharmacy Research Center, Tehran University of Medical Sciences, Tehran, Iran; e Medical Mycology of Razi Hospital, Tehran University of Medical Sciences, Tehran, Iran; f Razi Hospital Laboratory, Faculty member in medicine, Tehran University of Medical Sciences, Tehran, Iran

**Keywords:** Vaginitis, vaginal discharge, anti-candida

## Abstract

**Context:**

In Persian medicine, topical ingredients such as *Rosa damascena* Mill. (Rosaceae), are usually recommended for the treatment of uterine diseases. Scientific evaluation of these historical documents can be valuable for finding new potential use in conventional medicine.

**Objective:**

This clinical trial was performed to determine whether the use of the ‘ward’ vaginal tablet, which contains *Rosa damascena, Punica granatum* L. (Punicaceae), *Querqus infectoria* Oliv. (Fagaceae), *Myrtus communis* L. (Myrtaceae) and *Nardostachys jatamansi* (D.Don) DC. (Caprifoliaceae) could alleviate the symptoms of vulvovaginal candidiasis.

**Materials and methods:**

A parallel double-blinded placebo-controlled study was done. Eighteen to fifty-year-old women with vulvovaginal candidiasis were divided into the ‘ward’ and placebo groups, 46 individuals in each group. The ‘ward’ group received the ‘ward’ vaginal tablet containing 200 mg of dried extract. Placebo group received a placebo (composed of corn starch and lactose). One tablet was applied through the vagina for 7 consecutive nights.

**Results:**

Two weeks after medication administration, the vaginal discharge sample of patients was re-cultured; 29 patients (63.045%) in the ‘ward’ group and 6 (13.04%) patients in the placebo group had negative culture (*p* < 0.001). All clinical symptoms including itching, irritation, and vaginal discharge were significantly reduced in the ‘ward’ group compared with the placebo group following the intervention and the follow up (*p* < 0.05).

**Discussion and conclusions:**

The findings suggest the ‘ward’ vaginal tablet could ameliorate vulvovaginal candidiasis. Future larger studies are recommended due to compare the therapeutic effect of the ‘ward’ vaginal tablet with common treatments.

## Introduction

Vaginal infections recurrently persist that could increase women’s risk for sexually transmitted infections (McClelland et al. 2015). Vulvovaginal candidiasis (VVC) is described by vulvovaginal itching, redness, and discharge. *Candida albicans*, a common genitourinary tract commensal, has been the prominent species, which is the most common fungal agent isolated from clinical samples of patients suffering from VVC (Makanjuola et al. 2018). Roughly, three-quarters of all women are infected by VVC during their reproductive age; however, determining the exact incidence of VVC is not easy since many patients are self-treated (Dovnik et al. 2015). The diagnosis is regularly based completely on signs and symptoms with no tests for confirming the diagnosis (Achkar and Fries 2010). Treatment depends on whether the infection is complicated or uncomplicated (Pappas et al. 2009). Treating the complicated VVC is lengthy and most commonly involves multiple doses of oral fluconazole or at least 1 week of topical azoles (Dovnik et al. 2015); however, only fluconazole has been approved by the United States Food and Drug Administration (USFDA). These treatments fail to reduce the relapse rate of the disease, and their use is difficult due to the systemic and local side effects of azoles and contraindications in the first trimester of pregnancy (Boeke et al. 1993; Pendharkar et al. 2015). Due to these complications and the microbial resistance developed by use of these drugs (Richter et al. 2005), it is reasonable to suggest harmless, obtainable, well-tolerated, and effective drugs that are capable of decreasing the incidence of vaginal infections over prolonged periods (McClelland et al. 2015).

One of the options is the use of traditional drugs that have been popular among people for many years. In reliable Old Persian medicine references, such as the Canon of Medicine by ‘Avicenna’ (980–1037 AD), many herbal remedies including the ‘ward’ [Name of *Rosa damascena* Mill. (Rosaceae) in the Canon of Medicine] were recommended to treat vaginitis (Avicenna 2005). Considering the 10,000-year-old history of Persian medicine, the search for Iranian medical texts that have been used for centuries is a reasonable way to find new drugs, because the use of traditional experiences increases the likelihood of discovering effective drug substances by up to 40 times; this figure is only 1% by random research (Naseri et al. 2012). The anti-inflammatory, antifungal, and antimicrobial effects of these herbs and anti-*Candida* effect of *Rosa damascena, Punica granatum* L. (Punicaceae), and *Myrtus communis* L. (Myrtaceae) have been confirmed in numerous articles regarding conventional medicine (Kaur et al. 2004; Hayder et al. 2008; Boskabady et al. 2011; Hosseinzadeh et al. 2011; Shema-Didi et al. 2012; Anibal et al. 2013; Shin et al. 2015; Hosseini et al. 2016; Masoudi et al. 2017; Liu et al. 2018; Khalilzadeh et al. 2019). It is also recommended to use astringent and aromatic herbs in the treatment of vaginitis in Persian medicine resources (Azamkhan 2008). We investigated, in a randomised controlled trial, the influence of a vaginal tablet based on the ‘ward’ (*Rosa damascena)* on VVC (Table 1).

**Table 1.** Medicinal plants used for treatment of vaginitis mentioned in Persian medicine that exist in the ‘ward’ vaginal tablet.

Scientific name (s)	Family	Common name (s)	Persian name (s)	Herbarium code	Part used
*Rosa damascena*	Rosaceae	Damask rose	Gol-e-sorkh	PMP-553	Petals
*Nardostachys jatamansi*	Caprifoliaceae	Spikenard	Sonbol	PMP-276	Rhizome
*Myrtus communis*	Myrtaceae	Myrtle	Murd	PMP-443	Fruit
*Qerqus infectoria*	Fagaceae	Oak apple or Oak gall	Maazu	PMP-1675	Fruit
*Punica granatum*	Lythraceae	Pomegranate	Anaar	PMP-1674	Peel

## Materials and methods

### Study design

The topic of treatment of vaginal discharge was studied from the book ‘Exir Azam’, which summarised the views of Persian medicine scientists, and examined various formulas recommended as a vaginal tablet. Ultimately, this combination was selected based on the formula of the book ‘Exir Azam’ and vaginal astringent powder, which has been used for many types of vaginitis in Persian medicine clinics for many years (Azamkhan 2008).

The study used a randomised, placebo-controlled, double-blind clinical trial by applying a parallel design. In this study, the efficacy of the ‘ward’ vaginal tablet [composed of *Rosa damascena*, *Punica granatum, Myrtus communis, Querqus infectoria* Oliv. (Fagaceae) and *Nardostachys jatamansi* (D.Don) DC. (Caprifoliaceae)] on clinical and laboratory (culture and smear) symptoms of vulvovaginal candidiasis were compared against the placebo vaginal tablet.

### Plant material

Dried components of required plants [*Rosa damascena* petals (PMP-553), *Punica granatum* peel (PMP-1674), *Qerqus infectoria* fruit (PMP-1675), *Myrtus communis* fruit (PMP-443) and *Nardostachys jatamansi* rhizome (PMP-276)] were purchased from a local herbal drug market in October 2017. The sample was identified by Professor G. Amin and deposited at the Herbarium of the Faculty of Pharmacy, Tehran University of Medical Sciences (Table 1). Plant materials were powdered using a grinder. Also, polyethylene glycols, which were used in formulation, from Merck Company, were purchased.

### Extract preparation

Each powdered dried plant (with an equal weight ratio) was extracted for 72 h at room temperature (25 ± 2 °C) with hydro-alcoholic solvent (ethanol 70%) using the maceration method. The extract was condensed by a rotary evaporator. The dry weight ratio of this extract to the initial plant was 45%. The extract was kept at –18 ± 2 °C for the next experiments (Eftekhari et al. 2017).

### Total phenolic content

According to the Folin–Ciocalteu method with some changes, total phenolic compounds were evaluated. The ethanol extract (200 μL) was mixed with 1.5 mL of 10-fold diluted Folin–Ciocalteu reagent. The mixture was kept at 25 ± 2 °C for 5 min and then, 1.5 mL Na2CO3 7.5% solution was added. The mixture was also kept at 25 ± 2 °C. After 90 min, the absorbance level was evaluated at 725 nm using a UV-Visible spectrophotometer (GBC, Cintra 40). Total phenolic content was quantified by the calibration curve obtained by evaluating the absorbance of the known concentrations of Gallic acid standard solutions [10–150 μg/mL in 80% methanol]. The results were determined as Gallic acid equivalent (GAE) per 1 g dry powder and reported as mean value ± standard deviation (SD) (Hajimahmoodi et al. 2010).

### Preparing the vaginal tablet

Vaginal tablets containing 200 mg of dried extract were formulated by direct compression. The basal composition was lactose and corn starch in both the ‘ward’ vaginal tab and placebo. The weight of each tablet was 2 g. The prepared tablets were evaluated for colour, weight, and disintegration time according to Iranian pharmacopoeia standards. Also, the placebo was made with the appearance, colour, and physical properties of the ‘ward’ vaginal tablet. The tablets were prepared under the supervision of a specialist (Dr. Tayebeh Toliyat) in the pharmaceutical lab in the Faculty of Pharmacy, Tehran University of Medical Sciences, Tehran, Iran.

### Participants

Ninety-two women with VVC in Tehran, Iran, from March 2018 to October 2018, were enrolled in the study. The study protocol was according to the Declaration of Helsinki (2013 revision); the Ethics Committee of Tehran University of Medical Sciences approved the study [with reference number: IR.TUMS.VCR.REC.1396.3881]. The trial was registered in the Iranian Registry of Clinical Trials (registration ID: IRCT20171112037414N1). Also, all of the enrolled participants signed a written informed consent form.

The trial inclusion criteria were as follows:

- Married outpatient patients within the age range of 18–50 with signs of VVC (itching, irritation, vaginal edoema, dyspareunia, vaginal redness, and vaginal discharge) measured by visual analog scale (VAS), if the total score of the VAS scale for all symptoms was ≥4. To answer each question, the number 0 to 10 was set and the patient had to choose one number between 0 and 10, depending on the severity of symptoms. For example, if the patient gives score 2 to itching, and score 3 to dyspareunia, then sums it up to 5: thus, entering the study.
- Not having a pregnancy, lactation, and pregnancy intention during the study.
- Not using simultaneous treatments (chemical or herbal).
- Not suffering from chronic diseases such as immunocompromised, chronic hypertension and malignancies.

The exclusion criteria were as follows:

- If the score of the VAS scale for each of the symptoms was ≥6.
- Recurrent vulvovaginal candidiasis (RVVC).

The withdrawal criteria were as follows:

- Not taking the correct medicine.
- The patient unwillingness to continue treatment.
- Individuals requiring other treatment interventions.
- Any side effects caused by the drug.

### Laboratory procedures

Sample secretions of the upper part of the vaginal sidewall by sterile cotton swab were placed on two slides and one plate of the Sabro Dextrose Agar with Chloramphenicol medium. The specimen on the slide was then fixed after the flame was dried and the gram stain was applied. Examples were examined under a microscope with a magnification of forty. If the mycelium or blastospore was observed, the result of the microscopic evaluation was considered positive. The culture medium was evaluated for up to 72 h and was considered positive if the *Candida* colony was formed.

### Intervention

The enrolled patients were divided into two groups of the ‘ward’ and placebo based on randomisation using block randomisation method. Patients in the ward group received the ‘ward’ vaginal tablet (provided by Faculty of Pharmacy, Tehran University of Medical Sciences) one tablet for 7 consecutive nights. Patients in the placebo group were similar to the patients in the ‘ward’ group in terms of receiving vaginal starch powder tablets as a placebo in the same way and order. Starch powder tablet was packed in colour and containers like the ‘ward’ colour and containers.

### Randomisation

Ninety-two eligible patients were randomised in two parallel groups. A statistician generated a randomised list through the application of the simple block randomisation method. Then, the research assistant according to the randomised list assigned the eligible patients to two groups. All patients and physicians were blind to the way by which the patients were allocated.

### Outcome

The primary ending point of the study was the effect of the ‘ward’ vaginal tablet on VVC clinical features after 2 and 5 weeks compared to baseline. The secondary ending point was the side effects of this tablet after 2 weeks of treatment compared to baseline. In the clinical symptom form, we gave a score of 1–10 for 6 symptoms (mentioned in Table 2) including itching, irritation, vaginal discharge, dyspareunia, redness or edoema in the genital region with the VAS score, for each patient to select a number based on the severity of the symptoms.

**Table 2.** Comparison of mean, standard deviation and p value for study variables in two groups at 0, 2 and 5 weeks after start of the intervention.

	Baseline			Week 2			Week 5		
Parameter	Ward (*n* = 46)	Placebo (*n* = 46)	*p* value	Ward	Placebo	*p* value	Ward	Placebo	*p* value
Itching, Mean (SD)	3.07 (2.08)	2.41 (1.97)	0.101	0.52 (0.86)	1.87 (1.69)	<0.001	0.30 (0.76)	1.76(1.72)	<0.001
Irritation, Mean (SD)	2.02 (1.99)	1.83 (1.98)	0.641	0.24 (0.52)	1.33 (1.62)	<0.001	0.02 (0.15)	1.30 (1.60)	<0.001
Vaginal edoema, Mean (SD)	0.72 (1.53)	0.30 (0.94)	0.223	0.00 (0.00)	0.22 (0.70)	0.22	0.00 (0.00)	0.15 (0.60)	0.080
Dyspareunia, Mean (SD)	1.20 (1.87)	1.80 (2.10)	0.126	0.17 (0.44)	1.35 (1.75)	<0.001	0.07 (0.33)	1.26 (1.78)	<0.001
Vaginal redness, Mean (SD)	1.22 (1.79)	0.80 (1.53)	0.192	0.02 (0.15)	0.54 (1.09)	0.001	0.00 (0.00)	0.41 (1.00)	0.02
Vaginal discharge (by patient), Mean (SD)	4.39 (0.95)	3.65 (1.20)	0.070	0.89 (0.92)	3.26 (1.64)	<0.001	0.70 (1.11)	3.41 (1.60)	<0.001
Vaginal discharge (by physician), Mean (SD)	3.98 (0.95)	3.63 (1.20)	0.186	1.43 (0.86)	3.39 (1.00)	<0.001			
Smear, Number (%)	41 (89.13)	39 (84.78)	0.536	17 (36.96)	37 (80.43)	<0.001			
Culture, Number (%)	46 (100.00)	46 (100.00)	>0.999	17 (36.96)	40 (86.96)	<0.001			

Drug Complaint Questionnaire was prepared based on the Common Terminology Criteria for Adverse Events (CTCAE) Version 4.0. Evaluation of the possible side effects (such as itching, irritation, odourless discharge, vaginal dryness, vaginal pain, frequency, dyspareunia, redness or edoema in genital region) was done with short (yes/no) question on weeks two and five of the study and patients reports were logged. If the rate of any complication was similar to the symptom such as itching, irritation and discharge that had just occurred or increased in response to the drug, the drug side effects would have been considered.

### Statistical methods

Demographic data and basic clinical characteristics of the participants are shown as the mean ± standard deviation for continuous variables or number (percentage) for categorical variables. Statistical analysis was performed using Chi-square test for qualitative variables (Fisher exact test was used if the chi-square condition was not established) and the Mann–Whitney test was applied to compare two quantitative variables. The Wilcoxon Signed Ranks test was applied for the statistical analysis of the changes in vaginitis clinical and laboratory features before and after the interventions in each group. Since the parametric test assumptions were not established, alternative nonparametric tests were used. Statistical Package for the Social Sciences, version 15.0 (SPSS Inc., Chicago, IL, USA), was applied for statistical analyses. The significance level was set at 0.05.

## Results

### Determination of total phenolic content

As shown in Table 3, the total phenolic content of the mentioned plant extract and the formulation were reported in terms of gallic acid equivalent. The average phenolic percentage of the extract and tablet within three days was 17.03% and 15.73%, respectively.

**Table 3.** Total phenolic content of the extract and the ‘ward’ vaginal tablet in three different days.

	First day	Second day	Third day
Total phenolic content of the extract (mg/lit)	0.1706	0.1715	0.1689
	0.1697	0.1724	0.1695
	0.1702	0.1706	0.1694
Mean	0.1702	0.1715	0.1693
SD	0.343	0.706	0.245
Mean percentage of phenolic content of the extract (%)	17.02	17.15	16.93
Total phenolic content of the ‘ward’ vaginal tablet (mg/lit)	0.1589	0.1548	0.1579
	0.1593	0.1553	0.1581
	0.1586	0.1549	0.1583
Mean	0.1589	0.1550	0.1581
SD	0.238	0.216	0.208
Mean percentage of phenolic content of the ‘ward’ vaginal tablet (%)	15.89	15.50	15.81

### Baseline analysis

**Figure 1.**
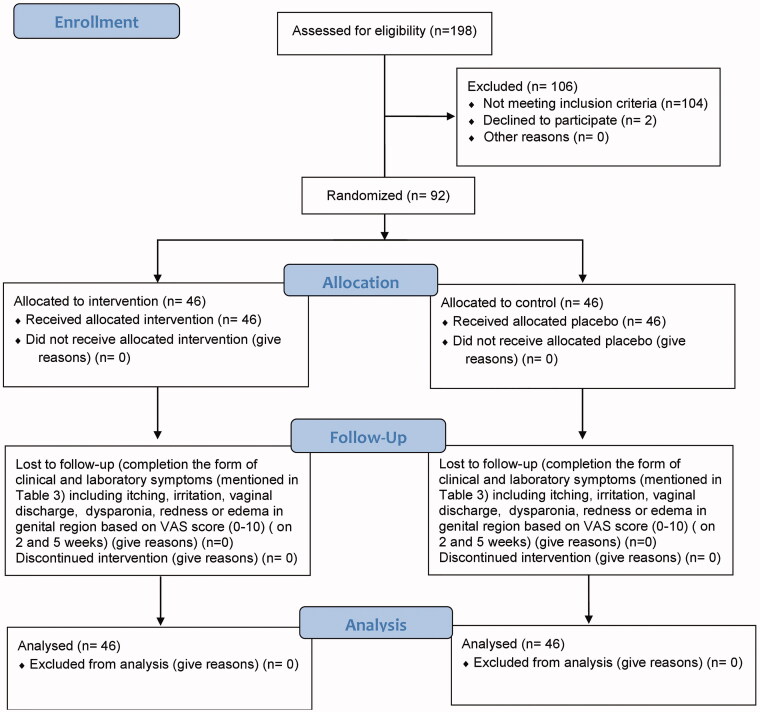
Flow chart of the study.

From March to October 2018, 198 volunteers were evaluated for eligibility. Ninety-two patients fulfilling the inclusion criteria agreeing to contribute to the trial were put into two groups. 46 patients were assigned to the ‘ward’ group and 46 to the placebo group. Figure 1 shows the detailed description of the patients’ enrolment, randomisation and follow up (CONSORT flowchart). Baseline characteristics were well balanced regarding age, body mass index, education, and job status (Table 4). The mean age of the participants was 35.43 ± 7.92 and 37.20 ± 9.36 years in the ‘ward’ and placebo groups, in the respective order. The two groups were not significantly different regarding the clinical and laboratory symptoms of vaginitis before the intervention.

**Table 4.** Demographic data and baseline clinical characteristics of the participants in the two groups of the study.

Parameter	Ward group (*n* = 46)	Placebo group (*n* = 46)	*p* value
Age (years), Mean (SD)	35.43 (7.92)	37.20 (9.36)	0.332
BMI (kg/m^2^), Mean (SD)	26.11 (3.72)	26.93 (5.02)	0.372
Education Number (%)			
Elementary	2 (4.35)	1 (2.17)	0.145
Cycle	1 (2.17)	5 (10.87)	
Diploma	18 (39.13)	16 (34.87)	
Associate degree	3 (6.52)	4 (8.70)	
Bachelor	14 (30.43)	19 (41.30)	
Master of science	5 (10.87)	1 (2.17)	
PhD	3 (6.52)	0 (0.0)	
Job status Number (%)			
Housewife	35 (76.09)	38 (82.61)	0.949
Coach	1 (2.17)	1 (2.17)	
Civil servant	5 (10.87)	3 (6.52)	
Teacher	2 (4.53)	2 (4.53)	
Physician	1 (2.17)	0 (0.00)	
Engineer	1(2.17)	0 (0.00)	
Scholar	1 (2.17)	1 (2.17)	
Beautician	0 (0.00)	1 (2.17)	

SD: standard deviation; BMI: body mass index, PhD: philosophiae doctor

### Efficiency

The mean age of participants was 36.32 years (standard deviation 8.66 years) and there was no significant difference between the two groups in terms of age, BMI, education and job status (*p* > 0.05). The most common complaints of patients at baseline were vaginal discharge (mean, [standard deviation] for the ward group = 4.39 [0.95] vs. placebo group = 3.65 [1.73]) (*p* = 0.070). 2 weeks after drug administration, the vaginal discharge of patients was re-cultured and 29 patients (63.045%) in the ‘ward’ group and 6 (13.04%) patients in placebo group had negative culture (*p* < 0.001). The ‘ward’ group and the placebo group were significantly different following the intervention and during the follow up (*p* < 0.05) (Table 2) and all studied clinical symptoms and mycelium or blastospore test were significantly improved after taking the ‘ward’ tablets. Clinical Cure Rate in the ‘ward’ group (10/46 × 100 = 21.73%) was significantly higher than the placebo group (1/46 × 100 = 2.17%) (*p* < 0.004) (Lorente et al. 2007). To evaluate its clinical curative effect the negative culture and absence of symptoms in weeks 5 was considered (Seidman and Skokos 2005; Fazel et al. 2012).

### Side effects

Possible side effects such as itching, irritation, odourless discharge or vaginal dryness, vaginal pain, frequency, dyspareunia, redness or edoema in the genital region evaluated on weeks two and five of the study and patients’ reports were logged. Most patients (82.61%) did not report any specific complications (*p*-value = 0.08). However, among the other registered complications, the main one was vaginal dryness (28.26%). The side effects of taking the drug or placebo were mild and minimised upon the completion of the drug (Table 5).

**Table 5.** Comparison of number, percentage and *p* value for side effects in two groups at 0 and 2 weeks after start of the intervention.

	Week 2		
Side effect	Ward (*n* = 46)	Placebo (*n* = 46)	*p* value
No side effect, Number (%)	26 (56.52)	38 (82.61)	0.080
Itching, Number (%)	7 (15.22)	2 (4.35)	
Irritation, Number (%)	0 (0.00)	1 (2.17)	
Odourless discharge, Number (%)	0 (0.00)	1 (2.17)	
Vaginal dryness, Number (%)	13 (28.26)	4 (8.70)	

## Discussion

In the present trial, the efficacy of the ‘ward’ vaginal tablet on clinical and laboratory symptoms of VVC was assessed through a double-blind randomised placebo-controlled clinical trial. Seemingly, the ‘ward’ vaginal tablet exerts significant effects on decreasing the symptoms of patients with VVC compared to the placebo group. According to the Persian medicine references, like Canon of Medicine by Avicenna, vaginal use of astringent plants such as ‘ward’ could alleviate the continuous outflow of moisture from the uterus due to uterine weakness in own food use; astringent plants, such as *R. damascena*, especially with strong scent increase the strength of the uterus and cause the contraction of uterus tissue and dry up secretions, so waste material cannot enter it (Avicenna 2005). As far as we know, this is the first clinical trial for assessing the effects of the ‘ward’ vaginal tablet on VVC. The most common complaints of patients at baseline were vaginal discharge (mean, [standard deviation] for the ward group = 4.39 [0.95] vs. placebo group = 3.65 [1.73]) (*p* = 0.070). Two weeks after drug administration, the vaginal discharge sample of patients was re-cultured and 29 patients (63.045%) in the ‘ward’ group and 6 (13.04%) patients in placebo group had negative culture (*p* < 0.001). All clinical and laboratory symptoms of vaginitis were significantly reduced in the ‘ward’ group compared with the placebo group following the intervention and the follow up (*p* < 0.05). The results suggested the ‘ward’ vaginal tablet to be effective in the treatment of VVC. Several *in vitro* and *in vivo* studies have reported the anti-inflammatory, antifungal, antimicrobial, and anti-*Candida* impacts of the plants applied in this vaginal tablet. The main mechanism causing vaginitis symptoms such as itching, irritation, and stimulation is inflammation by microorganisms (Khalilzadeh et al. 2019) and most studies have focussed on antioxidant and anti-inflammatory effects of plants we have used. The anti-inflammatory activity of the ‘ward’ vaginal tablet is probably involved in enhancing the symptoms of VVC in these patients. For example, *R. damascena* has been shown to have an anti-inflammatory effect. The effect of essential oil and hydro-alcoholic extract of *R. damascena* on rat paw edoema induced by carrageenan was demonstrated. *R. damascena* contains vitamin C, which has antioxidant and anti-inflammatory effects (Maleev et al. 1972; Tannenbaum et al. 1991; Hajhashemi et al. 2010). Also, the anti-inflammatory effect of Rosaceae is partly attributed to their abundance of phenolic compounds (Boskabady et al. 2011).

It is believed that the prostaglandins are also involved in the development of vaginitis (Khalilzadeh et al. 2019). The anti-inflammatory effect of the ‘ward’ vaginal tablet could be ascribed to its inhibitory activity on prostaglandin E2 (Pankevich et al. 2017). According to some studies, some plants of the ‘ward’ vaginal tablet or their phytochemicals showed the effects of PGE2 inhibition (Negi and Jayaprakasha 2003; Koeberle et al. 2009). The presence of analgesic agents is required to reduce the clinical symptoms of vaginitis, which was proved in several plants mentioned earlier (Babaee et al. 2010; Hosseinzadeh et al. 2011; Mo et al. 2013; Liu et al. 2018).

Pomegranate, myrtle, and oak are plants investigated in many clinical studies, and their efficacy was proved as well (Jacobs and Tomczak 2008; DiSilvestro et al. 2009; Babaee et al. 2010; Shema-Didi et al. 2012; Qaraaty et al. 2014; Ammar et al. 2016; Hosseini et al. 2016). A randomised clinical trial on *M. communis* (one of the components of the studied tablet) compared to metronidazole has shown the efficacy of this herb in bacterial vaginosis therapy (Masoudi et al. 2017).

Since several herbs have been used in this tablet, it is not known exactly which herbs are effective in this tablet. Another limitation was the short interval of the follow-up.

## Conclusions

Seemingly, the ‘ward’ vaginal tablet was capable of controlling all the clinical and laboratory symptoms of VVC. Future studies designed with higher sample sizes are recommended to confirm these results definitively.
